# The Soybean Gene *J* Contributes to Salt Stress Tolerance by Up-Regulating Salt-Responsive Genes

**DOI:** 10.3389/fpls.2020.00272

**Published:** 2020-03-17

**Authors:** Qun Cheng, Zhuoran Gan, Yanping Wang, Sijia Lu, Zhihong Hou, Haiyang Li, Hongtao Xiang, Baohui Liu, Fanjiang Kong, Lidong Dong

**Affiliations:** ^1^Innovative Center of Molecular Genetics and Evolution, School of Life Sciences, Guangzhou University, Guangzhou, China; ^2^Heilongjiang Academy of Agricultural Sciences, Mudanjiang, China; ^3^Institute of Farming and Cultivation, Heilongjiang Academy of Agricultural Sciences, Harbin, China; ^4^The Innovative Academy of Seed Design, Key Laboratory of Soybean Molecular Design Breeding, Northeast Institute of Geography and Agroecology, Chinese Academy of Sciences, Harbin, China

**Keywords:** Soybean, *J*, transcription factor, hairy roots, salt tolerance, RNA-seq

## Abstract

Soybean [*Glycine max* (L.) Merr.] is an important crop for oil and protein resources worldwide, and its farming is impacted by increasing soil salinity levels. In *Arabidopsis* the gene *EARLY FLOWERING 3* (*ELF3*), increased salt tolerance by suppressing salt stress response pathways. *J* is the ortholog of *AtELF3* in soybean, and loss-of-function *J*-alleles greatly prolong soybean maturity and enhance grain yield. The exact role of *J* in abiotic stress response in soybean, however, remains unclear. In this study, we showed that *J* expression was induced by NaCl treatment and that the J protein was located in the nucleus. Compared to NIL-*J*, tolerance to NaCl was significantly lower in the NIL-*j* mutant. We also demonstrated that overexpression of *J* increased NaCl tolerance in transgenic soybean hairy roots. J positively regulated expression of downstream salt stress response genes, including *GmWRKY12*, *GmWRKY27*, *GmWRKY54, GmNAC*, and *GmSIN1*. Our study disclosed a mechanism in soybean for regulation of the salt stress response. Manipulation of these genes should facilitate improvements in salt tolerance in soybean.

## Introduction

Soybean [*Glycine max* (L.) Merr.] is classified as a moderately salt-sensitive crop, and salt stress has negatively affected soybean yields (Parker et al., 1983; Ashraf, 1994; Munns and Tester, 2008). With increasing salinity levels, soybean production can be reduced by as much as 40% (Papiernik et al., 2005). Therefore, improving salt tolerance in soybean is essential to ensure future soybean yields. Some natural variations at the seedling stage in soybean have been identified through quantitative trait locus (QTL) mapping and genome-wide association studies (Lee et al., 2004; Chen et al., 2008; Hamwieh and Xu, 2008; Hamwieh et al., 2011; Ha et al., 2013; Guan et al., 2014; Patil et al., 2016; Zeng et al., 2017; Do et al., 2018). For instance, a major salt-tolerant QTL located on Chr.3 (linkage group N) has been identified repeatedly using different soybean-mapping populations (Lee et al., 2004; Hamwieh and Xu, 2008; Hamwieh et al., 2011; Ha et al., 2013). This QTL has been cloned with a whole-genome resequencing and map-based cloning approach and found to encode an ion transporter (Guan et al., 2014; Qi et al., 2014; Do et al., 2016). The function of this gene in NaCl tolerance was confirmed by using the transgenic hairy root and B2Y cell overexpression assay (Qi et al., 2014). Moreover, by using reverse genetics, several transcription factor (TF) genes and ion-exchanger genes have been identified to contribute to NaCl stress tolerance in soybean (Chen et al., 2014; Li et al., 2017; Xu et al., 2018). For instance, *GmWRKY27* encoded a WRKY TF and improved NaCl tolerance in transgenic soybean hairy roots (Wang et al., 2015). An NAC TF encoded by *SALT INDUCED NAC 1* (*GmSIN1*) and overexpression of *GmSIN1* promoted root growth and NaCl tolerance and increased yield under NaCl stress in soybean (Li et al., 2019). Ectopic expression of the *GmERF3* gene in transgenic tobacco plants gave tolerance to high salinity (Zhang et al., 2009). In addition, *GmCLC1* encoded Cl^–^/H^+^ antiporter and overexpression of *GmCLC1* enhanced NaCl tolerance in transgenic plants (Wei et al., 2016). However, few of circadian genes have been demonstrated to respond and adapt to high salinity.

*EARLY FLOWERING3* (*ELF3*) functions as one of the core circadian-clock components and was first determined to be a flowering repressor. For example, *elf3* mutants flower early in a photoperiod insensitive manner (Zagotta et al., 1996) and *ELF3*-overexpressing (*ELF3*-OX) plants bloom very late only under long-day conditions in Arabidopsis (Liu et al., 2001). In addition, ELF3 interacts with other circadian clock components, ELF4 and LUX, called the evening complex (Nusinow et al., 2011). This complex (ELF3-ELF4-LUX) binds to the promoters of *PIF4* and *PIF5* to repress hypocotyl growth in the evening (Nusinow et al., 2011). A recent report showed that *AtELF3*-OX plants are tolerant to high NaCl and that *elf3* mutants are hypersensitive to high NaCl in Arabidopsis (Sakuraba et al., 2017). Whether or not *AtELF3* homologous are involved in NaCl stress responses in soybean plant, however, remains largely unknown.

Our previous research showed that *J* is a co-ortholog of the Arabidopsis flowering-time gene *AtELF3* (Lu et al., 2017). However, whether this gene can respond to NaCl stress and the molecular mechanism, is largely unclear. In the present study, we demonstrated that expression of *J* was induced by NaCl and *J* protein was located in the nucleus. Transgenic soybean hairy roots overexpressing the *J* gene enhanced NaCl tolerance. *J* positively regulated the transcription levels of NaCl tolerance related genes *GmWRKY12*, *GmWRKY27*, *GmWRKY54*, *GmNAC11*, and *GmSIN1* in soybean, leading to NaCl stress tolerance. These studies allow for the elucidation of *J* roles in NaCl stress responses.

## Materials and Methods

### Plant Materials and NaCl Stress Treatment

Seedlings of soybean (NIL-*J* and NIL-*j* from Lu et al., 2017) were cultivated in a 8 × 8 cm flowerpot (vermiculite: nutritious soil is 1:3) and grown in a greenhouse under a photoperiod of 16 h light/8 h dark at 25°C and 60% humidity. For NaCl treatment, 12-day-old seedlings were watered with 200 mM sodium chloride (NaCl). For phenotype observations, we treated 12-day-old seedlings for 3 days.

### Measurements of Proline and Malondialdehyde Contents

Twelve-day-old NIL-*J* and NIL-*j* soybean seedings were watered with 200 mM NaCl treatment 2 days, leaves of NIL-*J* and NIL-*j* were harvested and immediately used. Both proline (Pro) and malondialdehyde (MDA) content were measured with the Pro assay kit (Yuanye, Shanghai, China, R30341) and MDA assay kit (Yuanye, R21870) based on the manufacturer’s protocols. All measurements were taken from three biological replicates.

### Quantitative PCR Analysis

For tissue-specific expression analyses, root, hypocotyl, cotyledon, leaf, stem, shoot apex, were collected from seedlings at first trifoliate (V1) stage, and flowers were collected from seedlings at first flowering (R1) stage. For total RNA extraction, leaf samples was harvested after 0, 1, 3, 6, 12, and 24 h of NaCl treatment, immediately frozen in liquid nitrogen, and stored at −80°C. Total RNA was isolated using TRIzol reagent (Invitrogen, Carlsbad, CA, United States, catalog number 15596018) and reverse-transcribed the total RNA according to the manufacturer’s instructions (Invitrogen). cDNA was synthesized from 1 μg of total RNA using a Super Script first-strand cDNA synthesis system (Takara, Dalian, China). Quantitative reverse transcription polymerase chain reaction (qRT-PCR) analysis was performed to measure *J* transcription levels on a Roche LightCycler480 system (Roche, Mannheim, Germany) using a real-time PCR (RT-PCR) kit (Roche). Briefly, the cDNA was diluted to 10-fold and used 1 μL of diluted cDNA as the template in a 20 μL qPCR reaction, which was predenatured at 95°C for 5 min, followed by a 40-cycle program (95°C, 10 s; 60°C, 10 s; 72°C, 20 s per cycle). The soybean housekeeping genes *GmTUB* (*Glyma.05G157300*) (Cheng et al., 2019) and *GmEF1*β (*Glyma.17G001400*) (Jian et al., 2008) were used as an internal reference for normalization. The relative transcription level of the target gene was calculated using the 2^–Δ^ ^Δ^ ^*CT*^ method. We used three biological replicates and three technical repeats in all assays.

### Subcellular Localization of the *J-GFP* Fusion Proteins

The coding sequence of *J* was amplified by RT-PCR using primers *J-GFPF* and *J-GFPR* Supplementary Table S1, fused to the N-terminus of green fluorescent protein (GFP) under the control of the constitutive Cauliflower Mosaic Virus 35S (CaMV35S) promoter. The resulting expression vector, *p35S*:*J*-*GFP*, was inserted into *A. tumefaciens* strain GV3101 cells, and transfected into healthy leaves of 21-d-old *Nicotiana benthamiana* (*N. benthamiana*) tobacco leaves by agroinfiltration as described previously (Cheng et al., 2018). The fluorescence signals were imaged using an LSM800 spectral confocal microscope imaging system (Zeiss, Oberkochen, Germany). The *p35S*-*GFP* vector was used as a control.

### *Agrobacterium rhizogenes*–Mediated Transformation of Soybean Hairy Roots

The full-length coding sequence of *J* from Harosoy was cloned into the pTF101-Gene vector (containing the bar gene for glufosinate resistance), between *Avr*II and *Mlu*I sites downstream of the constitutive CaMV35S promoter. As a negative control, the gene for the *GFP* was cloned and instead of *J* using the same vector and promoter. Both constructs (*p35S*-*J* and *p35S*-*GFP*) were introduced into *Agrobacterium rhizogenes* strain K599. Soybean hairy root transformation was performed as previously described by Cheng et al. (2018) with some modifications. Surface-sterilized soybean seeds were germinated on a germination medium [3.21 g/L Gamborg Basal salt mixture (Gamborg et al., 1968), 1.0 mg/L 6-BA, 2% sucrose, 0.8% agar, pH 5.8] for 5 days (16 h light/8 h dark). *Agrobacterium rhizogenes* strain K599 containing the recombinant construct was grown in yeast extract peptone medium containing 50 mg/L kanamycin and 25 mg/L rifampicin at 28°C for 16 h. We then used the construct to infect the cotyledons through scalpel incisions. The cotyledons were co-cultivated with *A. rhizogenes* on root-inducing medium [4.3 g/L Murashige and Skoog (MS) medium (Murashige and Skoog, 1962), 3% sucrose, 0.6 g/L MES, 250 mg/L cefotaxime and 250 mg/L carbenicillin]. After 2 weeks, cotyledons with roots emerging from the incision sites were transferred to new root-inducing medium with NaCl or medium without NaCl as untreated control. Root mass was weighed about 1 week after treatment and used the soybean plant NIL-*j* for transformation. The overexpression of the *J* gene was tested in transgenic hairy roots using qRT-PCR.

### Transcriptomic Analysis

NIL-*J* and NIL-*j* soybean plants grown for 4 weeks under non-stress conditions were used for transcriptomic analysis. Total RNA was extracted from the samples with three biological replications using the Spectrum Plant Total RNA Kit (Sigma-Aldrich, St. Louis, MO, United States, STRN10-1KT). The sequencing libraries were generated using NEB Next Ultra RNA Library Prep Kit for Illumina (New England Biolabs, Ipswich, MA, United States) following the manufacturer’s recommendations and added index codes to attribute sequences to each sample. The clustering of the index-coded samples was performed on a cBot Cluster Generation System using TruSeq PE Cluster Kit v4-cBot-HS (Illumia) according to the manufacturer’s instructions. After cluster analysis, we sequenced the RNA on an Illumina Hiseq 2500 platform to generate paired-end reads. We mapped the total reads to the soybean genome^1^ using the Tophat tools software (Trapnell et al., 2009). Read counts for each gene were generated using HTSeq with a union mode. Differentially expressed genes (DEGs) among samples were defined by DESeq using two separate models (Anders and Huber, 2010), based on fold change greater than two and a false discovery rate (FDR)–adjusted *P* value < 0.05. We implemented gene ontology (GO) enrichment analysis of the DEGs using the GOseq R packages based on Wallenius non-central hypergeometric distribution (Young et al., 2010), which can adjust for gene length bias in DEGs.

### Statistical Analyses

For phenotypic evaluation, we analyzed at least 10 NIL-*J* and NIL-*j* soybean plants, or *GFP-OE* and *J-OE* transgenic hairy roots. The exact numbers of individuals (*n*) are presented in the figure legends. For expression analyses using qRT-PCR, we pooled at least three individuals per tissue sample and performed at least three qRT–PCR reactions (technical replicates). The exact number of replicates is given in the figure legends. We compared mean values for each measured parameter using one-way analysis of variance from SPSS (version 20, IBM, Chicago, IL, United States) or one-tailed, two-sample Student’s *t* tests from Microsoft Excel, whenever appropriate. The statistical tests used for each experiment were given in the figure legends.

## Results

### *J* Gene Expression and Protein Localization

**FIGURE 1:**
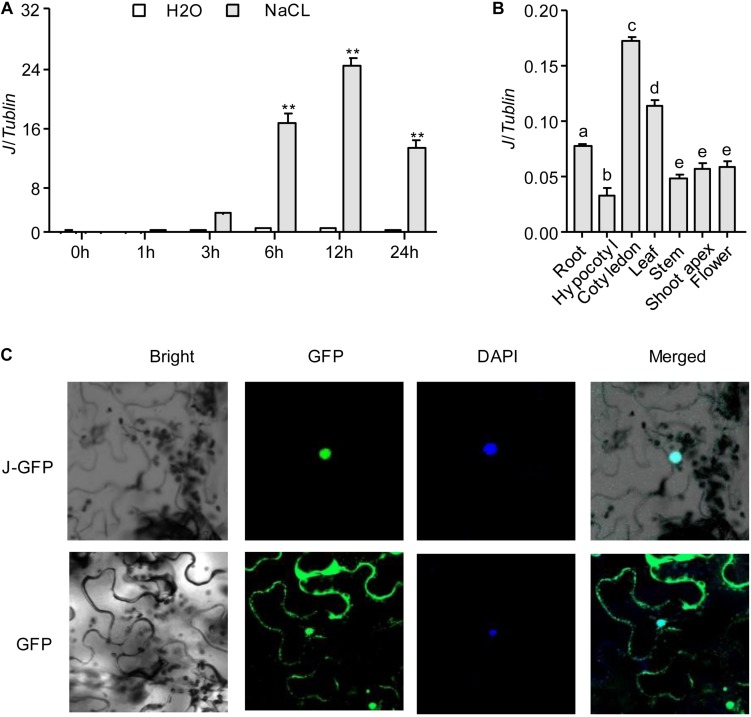
*J* gene expression and protein localization. **(A)** *J* expression levels in response to NaCl treatment in soybean seedlings as revealed by qRT-PCR analysis. Significant differences were analyzed based on the results of three biological replications (Student’s *t* test: **P < 0.01). Bars indicate standard error of the mean. The presence of the same lowercase letter above the histogram bars in a–e denotes non-significant differences across the two panels (*P* > 0.05). **(B)** *J* expression in various organs of soybean plants. **(C)** Subcellular localization of *J* protein in in tobacco leaf cells. DAPI, fluorescence of 4′,6-diamidino-2-phenylindole; Merge, merge of GFP and DAPI.

Our previous research showed that *J* is a co-ortholog of the Arabidopsis flowering-time gene *AtELF3.* J promotes flowering of soybean by directly repressing the expression of *E1* (Lu et al., 2017). To understand whether *J* was involved in the response to NaCl stress in soybean, we first investigated the expression of *J* in soybean seedlings exposed for 2 weeks to NaCl (200 mM). The results showed that *J* expression was significantly induced and reached a peak at 12 h under NaCl exposure (Figure 1A). We next investigated the expression pattern of *J* by quantifying the relative abundance of the mRNA in different organs. *J* was constitutively expressed in soybean organs (root, hypocotyl, cotyledon, leaf, stem, shoot apex, flower) and highly expressed in the cotyledons, but it was expressed moderately in leaves and roots (Figure 1B). We further determined the subcellular localization of *J.* The *p35S-J*-*GFP* construct was transiently transformed into *N. benthamiana* leaf cells. The results show that *J* is located in the nucleus, and the GFP control is located primarily in the cytoplasm (Figure 1C and Supplementary Figure S1). These results indicate that *J* is a nuclear protein, and that the expression of *J* is induced by NaCl treatment.

### *J* Improves Salt Tolerance in Soybean

**FIGURE 2:**
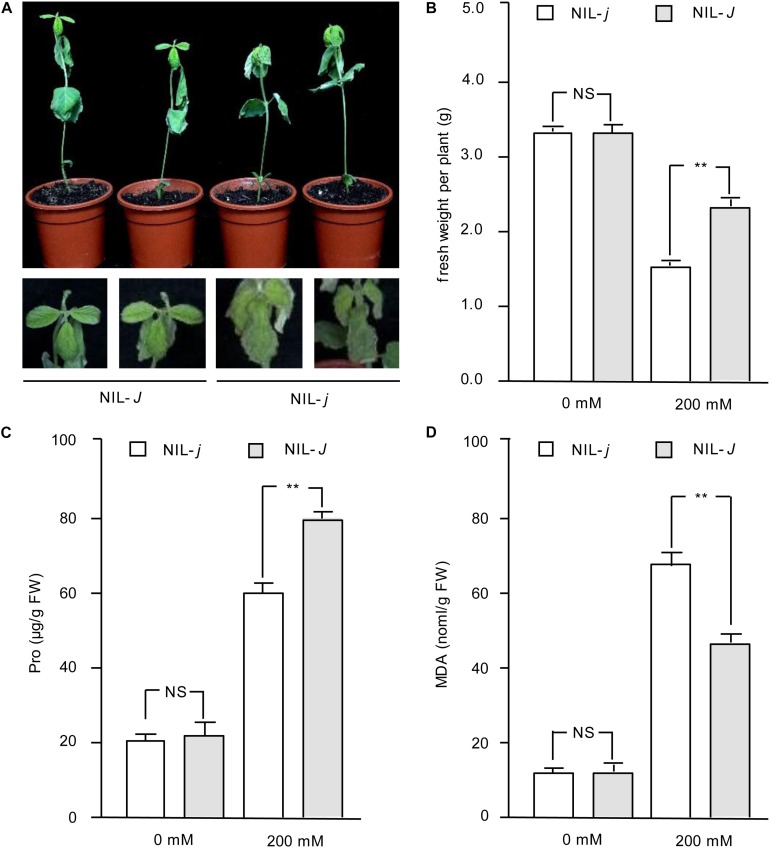
Phenotype identification of *J* under NaCl treatment in NIL-*J* and NIL-*j* soybean plants. **(A)** Phenotypes of 14 days NIL-*J* and NIL-*j* seedings after planting, treated with 200 mM NaCl for 3 days, Bottom photos: second leaves. *N* = 12. **(B)** Fresh weight of NIL-*J* and NIL-*j* soybean plants with 0 mM or 200 mM NaCl treatment. **(C)** Proline contents in NIL-*J* and NIL-*j* soybean seedlings under 0 mM or 200 mM NaCl treatment. **(D)** MDA contents in NIL-*J* and NIL-*j* soybean seedlings under 0 mM or 200 mM NaCl treatment. Error bars, s.e.m. Data were analyzed using Student’s *t* test. NS, not significant. **P < 0.01.

Because the expression of *J* was induced under NaCl treatment, we hypothesized the *J* gene may have a role in salt tolerance in soybean. To confirm this potential function, we examined seedlings from near-isogenic lines (NILs) carrying the functional *J* allele (NIL-*J*) or the non-functional *j* allele (NIL-*j*) (Lu et al., 2017) for their sensitivity to 200 mM NaCl. NIL-*j* seedlings were severely wilted and almost 99% of the leaves exhibited serious dehydration and drying (Figure 2A). Although old leaves of NIL-*J* soybean seedlings wilted, new leaves still grew vigorously (Figure 2A). The fresh weight was measured under NaCl treatment, and the results showed that the fresh weight of NIL-*J* soybean seedlings was significantly higher than that of NIL-*j* plants (Figure 2B). Next, we measured MDA and Pro content to compare stress impact between NIL-*J* and NIL-*j*. The results showed that NIL-*J* soybean seedlings increased Pro content to a larger extend than the NIL-*j* lines (Figure 2C), whereas the MDA content was less increased in the NIL-*J* lines under NaCl stress (Figure 2D). These measurements suggest the impact of the NaCl treatment is lower in the NIL-*J* lines.

**FIGURE 3:**
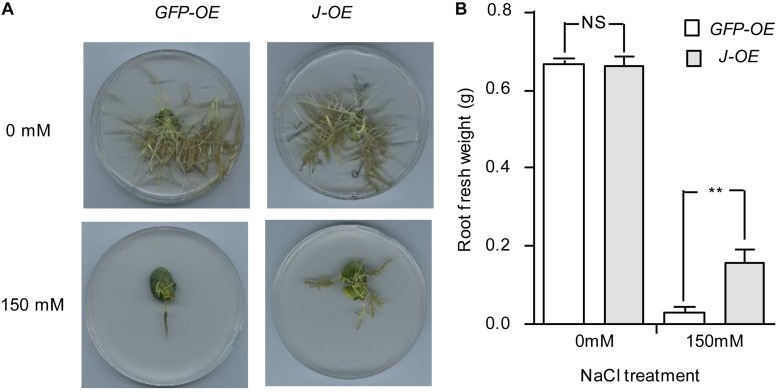
Phenotype identification of *J* under salt treatment in transgenic hairy roots. **(A)** Phenotypes of transgenic hairy roots expressing either GFP or *J* with or without NaCl treatment. Photos were taken 2 weeks after treatment. **(B)** Fresh weight of hairy roots with or without NaCl treatment. *N* = 12. Error bars, s.e.m. Data were analyzed using Student’s *t* test. NS, not significant. **P < 0.01.

To further evaluate whether *J* is a NaCl-tolerant gene, a construct for *J* overexpression (*pTF101*-*J*) was generated and transformed into the soybean hairy roots of NIL*-j*. We confirmed the expression of the transgene by qRT-PCR (Supplementary Figure S1). In the absence of NaCl treatment, both root cultures transformed with either *J* or green fluorescent protein (*GFP*; control) gave healthy hairy roots (Figure 3A). When subjected to NaCl treatment, however, roots transformed with *J* showed significantly higher root fresh weights than the control (Figure 3B). This result support the idea that *J* could reduce NaCl stress.

### Transcriptomic Analysis of NIL-*J* and NIL-*j* Soybean Plants

**FIGURE 4:**
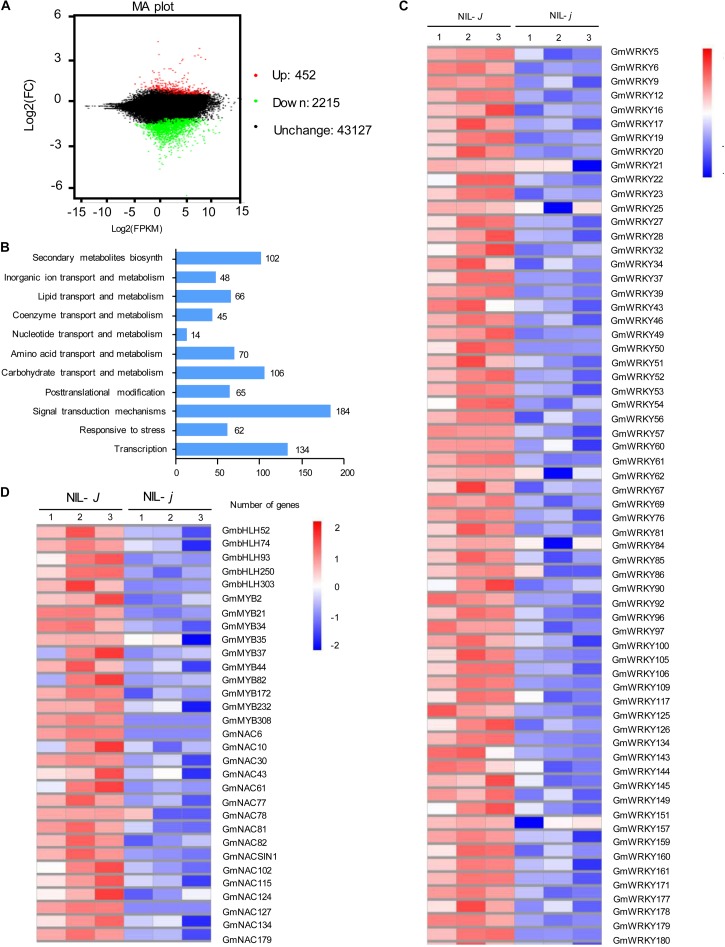
Transcriptomic analysis of NIL-*J* and NIL-*j* soybean plant. **(A)** Numbers of genes showing differential expression between NIL-*J* and NIL-*j* soybean plant in non-NaCl-stressed seedings. **(B)** GO terms that were statistically enriched in differentially expressed genes in NIL-*J* and NIL-*j* RNA-seq assay. The numbers near the columns indicate the number of differentially expressed genes. **(C,D)** The heat map of differential expression of WRKY, bHLH, MYB, and NAC family genes in NIL-*J* and NIL-*j*. The numerical values for the blue-to-red gradient bar represent log2-fold change relative to the control sample.

To identify genes possibly related to the *J*-mediated reduction of NaCl impact, we performed mRNA-sequence (RNA-Seq) analysis of the full transcripts from NIL-*J* and NIL-*j* soybean plants. We identified 2567 DEG that were affected more than two-fold in NIL-*j* compared with NIL-*J* under non-stress conditions (FDR *P* < 0.05; Figure 4A and Supplementary Data S1). Among the 2567 DEG, 452 genes were significantly upregulated and 2115 genes were significantly downregulated (Figure 4A and Supplementary Data S1). The GO terms specifically enriched in the downregulated DEGs were primarily genes involved in stress responses, in transcription, in secondary metabolite biosynthesis, in transport of organic ions, and signal transduction (Figure 4B).

Biotechnological and RNA-Seq approaches have identified some TF families, such as WRKY, NAC, MYB, and bHLH proteins, that respond to NaCl stress in soybean. Here, we found that 64 WRKY-family genes, 16 NAC-family genes, 10 MYB-family genes, and 5 bHLH-family genes were significantly downregulated in NIL-*j* plants in comparison to NIL-*J* (fold-change > 2, and *P* > 0.5) under normal conditions (Figures 4C,D). To further explore the effect of *J* on the transcription of NaCl related genes, we determined, for all of these genes, whether they respond to NaCl stress. As a result, we identified that 24 of 64 WRKY-family genes and 2 of 16 NAC-family genes that could respond to NaCl stress in soybean (Table 1). Therefore, we speculated that *J* may positively regulate the expression of these genes and contribute to improvements in NaCl tolerance in soybean.

**TABLE 1:** *J* up-regulating genes confer salt tolerance in soybean.

**Gene name**	**Gene number**	**Function**	**References**
*GmWRKY12*	Glyma.01G224800	Improve salt tolerance in soybean	Shi et al., 2018
*GmWRKY20*	Glyma.11G163300	Response to salt stress in soybean	Yu et al., 2016
*GmWRKY21*	Glyma.04G218700	Response to salt stress in soybean	Yu et al., 2016
*GmWRKY27*	Glyma.15G003300	Improve salt tolerance in soybean	Wang et al., 2015
*GmWRKY28*	Glyma.01G056800	Response to salt stress in soybean	Yu et al., 2016
*GmWRKY50*	Glyma.04G076200	Response to salt stress in soybean	Yu et al., 2016
*GmWRKY51*	Glyma.03G220800	Response to salt stress in soybean	Yu et al., 2016
*GmWRKY54*	Glyma.19G094100	Improve salt tolerance in *Arabidopsis*	Zhou et al., 2008
*GmWRKY56*	Glyma.08G218600	Response to salt stress in soybean	Yu et al., 2016
*GmWRKY57*	Glyma.18G213200	Response to salt stress in soybean	Yu et al., 2016
*GmWRKY62*	Glyma.18G056600	Response to salt stress in soybean	Yu et al., 2016
*GmWRKY76*	Glyma.03G042700	Response to salt stress in soybean	Yu et al., 2016
*GmWRKY81*	Glyma.04G061400	Response to salt stress in soybean	Yu et al., 2016
*GmWRKY85*	Glyma.04G238300	Response to salt stress in soybean	Yu et al., 2016
*GmWRKY92*	Glyma.05G184500	Response to salt stress in soybean	Song et al., 2016
*GmWRKY100*	Glyma.06G168400	Response to salt stress in soybean	Yu et al., 2016
*GmWRKY125*	Glyma.09G274000	Response to salt stress in soybean	Yu et al., 2016
*GmWRKY126*	Glyma.09G280200	Response to salt stress in soybean	Yu et al., 2016
*GmWRKY134*	Glyma.13G117600	Response to salt stress in soybean	Yu et al., 2016
*GmWRKY144*	Glyma.14G103100	Response to salt stress in soybean	Song et al., 2016
*GmWRKY159*	Glyma.17G011400	Response to salt stress in soybean	Yu et al., 2016
*GmWRKY171*	Glyma.18G208800	Response to salt stress in soybean	Yu et al., 2016
*GmWRKY179*	Glyma.19G254800	Response to salt stress in soybean	Yu et al., 2016
*GmWRKY180*	Glyma.20G028000	Response to salt stress in soybean	Yu et al., 2016
*GmNAC11*	Glyma.19G108800	Improve salt tolerance in *Arabidopsis*	Hao et al., 2011
*GmSIN1*	Glyma.13G279900	Improve salt tolerance in soybean	Li et al., 2019

### *J* Improves Salt Tolerance by Positively Regulating Salt Stress Response Genes

**FIGURE 5:**
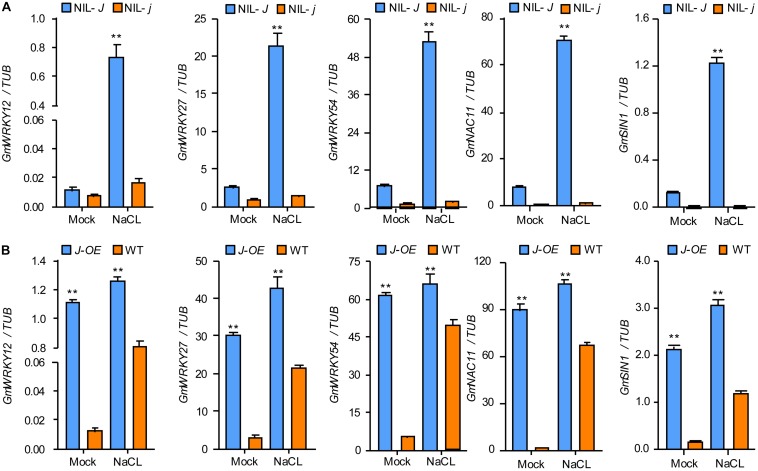
*J* positively regulating the expression of salt stress–tolerant genes in soybean. **(A)** The transcription levels of *GmWRKY12, GmWRKY27*, *GmWRKY54*, *GmNAC11*, and *GmSIN1* in 15-day-old seedlings of NIL-*J* and NIL-*j* soybean plant exposed to either 0 mM (mock) or 150 mM NaCl for 6 h; data obtained by qRT-PCR. **(B)** The transcription levels of *GmWRKY12*, *GmWRKY27*, *GmWRKY54*, *GmNAC11*, and *GmSIN1* in *J* or *GFP* (Control) overexpressing soybean hairy root and exposed to either 0 mM (mock) or 150 mM NaCl; data obtained by qRT-PCR. Significant differences were analyzed based on the results of three biological replications (Student’s *t-*test: **P < 0.01). Bars indicate standard error of the mean.

The comparison of the transcriptomes of 12 d old NIL-*J* and NIL-*j* soybean seedlings, showed higher expression of *GmWRKY12*, *GmWRKY27*, *GmWRKY54*, *GmNAC11*, and *GmSIN1* in NIL-*J* lines. To confirm these differential expressions, and simultaneously test expression changes under NaCl treatment, we used qRT-PCR in NIL-*J* and NIL-*j* soybean plants and in *J*-overexpressing (*J-OE*) soybean hairy roots. These genes were all upregulated in NIL-*J* soybean plants (Figure 5A and Supplementary Figure S2) and *J-OE* soybean hairy roots (Figure 5B and Supplementary Figure S2). Additionally, they all showed earlier or higher induction in NIL-*J* than NIL-*j* soybean plants or in *J-OE* hairy roots than in WT plants in response to NaCl (Figures 5A,B and Supplementary Figure S2). These data suggested that *J* expression regulates to some extend the expression of *GmWRKY12*, *GmWRKY27*, *GmWRKY54*, *GmNAC11*, and *GmSIN1* and can improve NaCl tolerance in soybean.

## Discussion

To engineer salt-tolerant soybean varieties, it is crucial to identify key components of the plant salt-tolerance network. Although some salt-tolerance genes have been identified in soybean, knowledge about the mechanisms by which they work is still scarce. In this study we investigated the potential role and mechanism for one such candidate, named *J*, for which the Arabidopsis-ortholog *AtELF3* may be involved in stress responses (Lu et al., 2017). Recently, research showed that AtELF3 enhances the resilience to NaCl stress and plays a key role in the repression of ROS production under NaCl stress in Arabidopsis (Sakuraba et al., 2017). Consistent with these observations, we demonstrated that *J* improved NaCl tolerance in soybean plants. This finding suggested that the *ELF3* homologous gene may have a similar function in response to NaCl stress in other crops.

It has been reported that WRKY family TFs play an important role in response to NaCl stress in soybean (Zhou et al., 2008; Wang et al., 2015; Song et al., 2016; Shi et al., 2018; Xu et al., 2018). Zhou et al. (2008) identified 64 *GmWRKY* genes before the soybean genome was sequenced and confirmed that *GmWRKY13*, *21* and *54* genes were involved in NaCl stress. Yu et al. (2016) identified 188 soybean WRKY genes genome-wide, and 66 of the genes have been shown to respond rapidly and transiently to the imposition of NaCl stress. In the latest version of the soybean genome (Wm82.a2v1), 176 GmWRKY TFs were identified and the expression of three *GmWRKY* genes increased under NaCl treatment (Song et al., 2016). In addition, some NAC TFs have been involved in NaCl stress responses (Hao et al., 2011; Melo et al., 2018; Li et al., 2019). For example, overexpression of *GmNAC11* resulted in enhanced tolerance to NaCl stress (Hao et al., 2011). In this study, we found that J upregulated 64 WRKY-family genes and 16 NAC-family genes by transcriptomic analysis. Based on RNA-Seq and bioinformatics methods, we found that 24 WRKY-family genes and two NAC-family genes may have participated in response to NaCl in soybean. We also confirmed that J positively regulated the expression of *GmWRKY12*, *GmWRKY27*, *GmWRKY54*, *GmNAC11*, and *GmSIN1*, which encoded a positive effect on NaCl tolerance in soybean (Zhou et al., 2008; Hao et al., 2011; Wang et al., 2015; Shi et al., 2018; Li et al., 2019). AtELF3 participated in the evening (AtELF3-AtELF4-AtLUX) complex of the transcriptional repression of downstream genes (Nusinow et al., 2011). A recent study revealed that AtELF3 indirectly binds to the *AtPIF4* promoter and represses the expression of *AtPIF4*. AtPIF4 directly downregulates the transcription of *JUNGBRUNNEN1* (*JUB1/ANAC042*), encoding a TF that upregulates the expression of NaCl stress–tolerant genes (Sakuraba et al., 2017). Thus, we speculated that *J* may indirectly regulate the transcription of *GmWRKY* and *GmNAC* genes, which positively regulated NaCl stress response pathways in soybean. In future work, we will identify whether or not *J* directly regulates genes in soybean NaCl stress response pathways.

Overall, our results showed that *J* transcription was activated under NaCl stress in soybean. and *J* could positively regulate the expression of salt-responsive genes in soybean. Our findings indicate that J may function in plant survival under high NaCl levels, and may provide a target for genetically designing and breeding of more salt-tolerant soybean.

## Data Availability Statement

The datasets generated by this study can be found in the NCBI using accession number PRJNA605480.

## Author Contributions

LD, FK, and BL designed the experiments and managed the projects. QC, ZG, and YW performed the experiments. SL, ZH, HL, and HX performed the data analysis. LD and QC wrote the manuscript.

## Conflict of Interest

The authors declare that the research was conducted in the absence of any commercial or financial relationships that could be construed as a potential conflict of interest.

## Footnotes

1 https://phytozome.jgi.doe.gov/pz/portal.html

## Supplementary Material

FIGURE S1 Expression of transgenes validated by qRT-PCR. **(A)** Transgenic hairy root expressing of *GFP*. **(B)** Transgenic hairy root expressing of *J.* Significant differences were analyzed based on the results of three biological replications (Student’s t test: ***P* < 0.01). Bars indicate standard error of the mean. N ≥ 12. Error bars = s.e.m.

FIGURE S2 J positively regulating the expression of NaCl stress tolerance genes in soybean. **(A)** The transcript levels of *GmWRKY12*, *GmWRKY27*, *GmWRKY54*, *GmNAC11*, and *GmSIN1* in Fifteen-day-old seedlings of NIL-*J* and NIL-*j* soybean plant exposed to either 0 mM (mock) or 150 mM NaCl for 6 h; data obtained by qRT-PCR. **(B)** The transcript levels of *GmWRKY12*, *GmWRKY27*, *GmWRKY54*, *GmNAC11*, and *GmSIN1* in *J* or *GFP* (Control) overexpressed soybean hairy root and exposed to either 0 mM (mock) or 150 mM NaCl; data obtained by qRT-PCR. All data were normalized to levels of amplified soybean EF1β. Significant differences were analyzed based on the results of three biological replications (Student’s t test: ***P* < 0.01). Bars indicate standard error of the mean.

TABLE S1 Primers used for this study.

DATA S1 List of genes with significant expression changes.
