# Supplementary figures and images for: The Soybean Gene J Contributes to Salt Stress Tolerance by Up-Regulating Salt-Responsive Genes
Source: Front Plant Sci. 2020 Mar 17;11:272. doi: 10.3389/fpls.2020.00272 (PMC7090219; doi:10.3389/fpls.2020.00272)

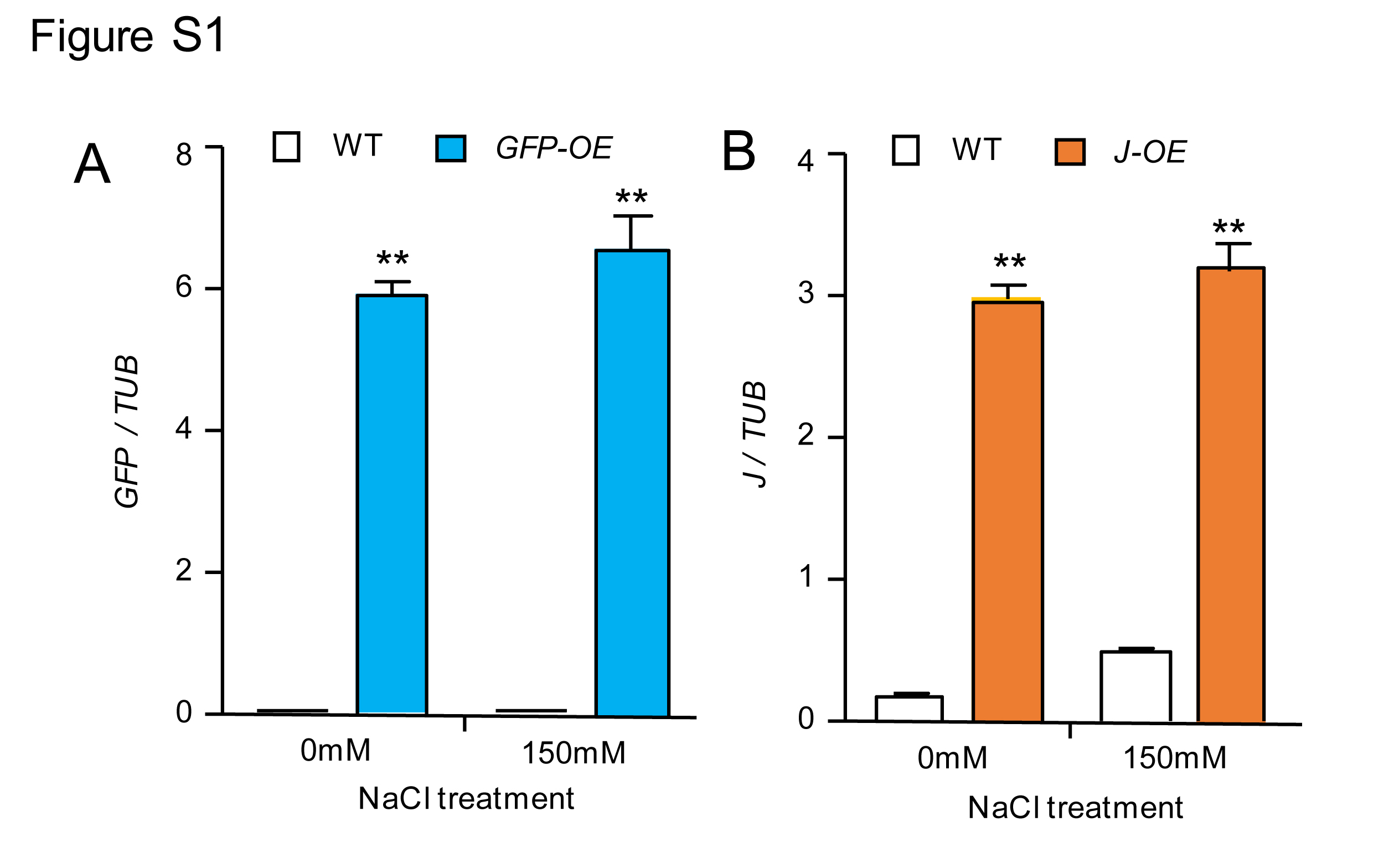

Supplement: FIGURE S1 — Expression of transgenes validated by qRT-PCR. (A) Transgenic hairy root expressing of GFP. (B) Transgenic hairy root expressing of J. Significant differences were analyzed based on the results of three biological replications (Student’s t test: **P < 0.01). Bars indicate standard error of the mean. N ≥ 12. Error bars = s.e.m. [file Image_1.JPEG]

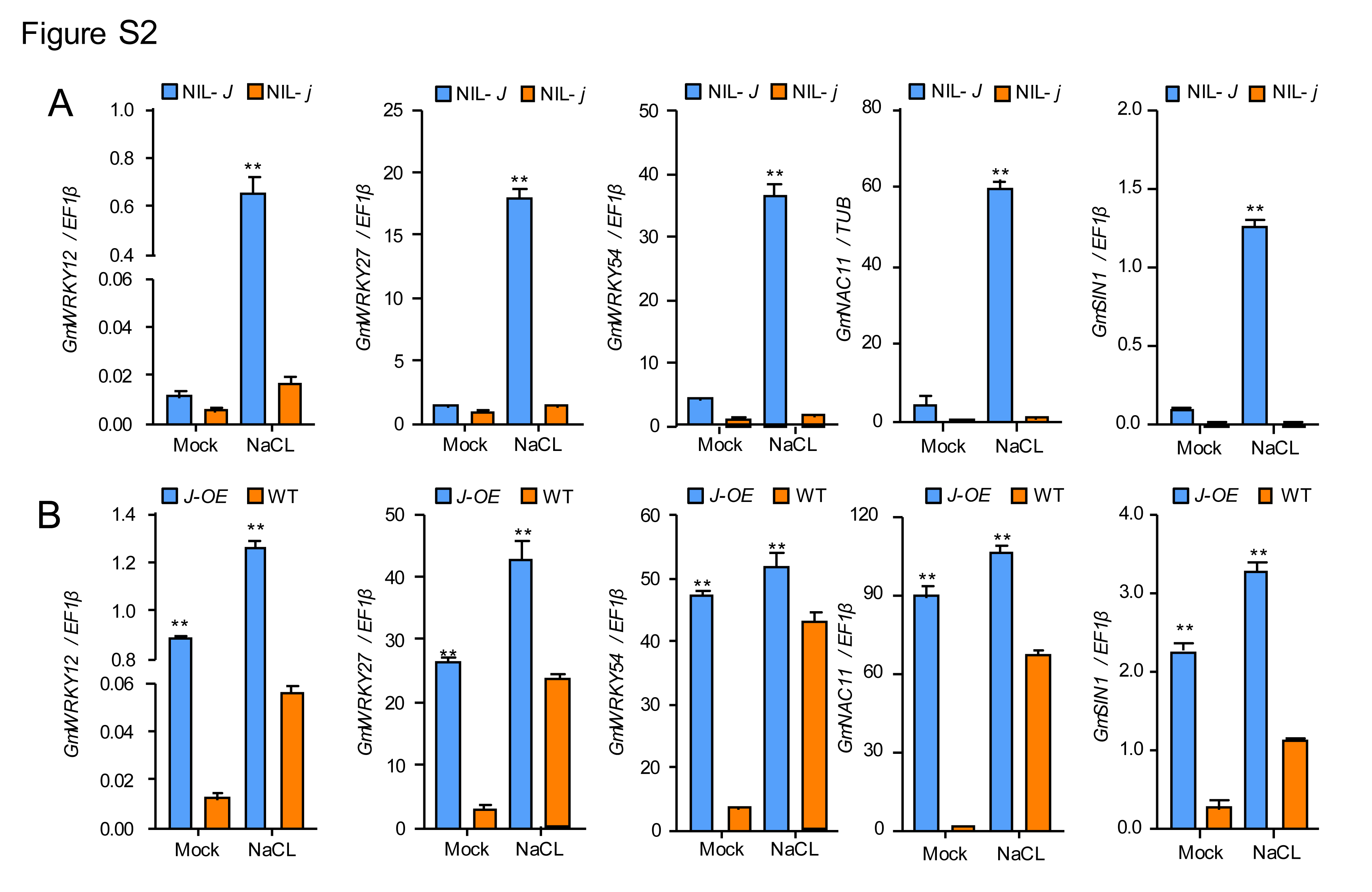

Supplement: FIGURE S2 — J positively regulating the expression of NaCl stress tolerance genes in soybean. (A) The transcript levels of GmWRKY12, GmWRKY27, GmWRKY54, GmNAC11, and GmSIN1 in Fifteen-day-old seedlings of NIL-J and NIL-j soybean plant exposed to either 0 mM (mock) or 150 mM NaCl for 6 h; data obtained by qRT-PCR. (B) The transcript levels of GmWRKY12, GmWRKY27, GmWRKY54, GmNAC11, and GmSIN1 in J or GFP (Control) overexpressed soybean hairy root and exposed to either 0 mM (mock) or 150 mM NaCl; data obtained by qRT-PCR. All data were normalized to levels of amplified soybean EF1β. Significant differences were analyzed based on the results of three biological replications (Student’s t test: **P < 0.01). Bars indicate standard error of the mean. [file Image_2.JPEG]
