# Supplementary material for: The Soybean Gene J Contributes to Salt Stress Tolerance by Up-Regulating Salt-Responsive Genes
Source: Front Plant Sci. 2020 Mar 17;11:272. doi: 10.3389/fpls.2020.00272 (PMC7090219; doi:10.3389/fpls.2020.00272)
Supplement: TABLE S1 — Primers used for this study. [file Table_1.docx]

**Supplemental Table 1.** List of Primers Used in This Study.

| **Primer name** | **Primers (5’-3’)** |
| --- | --- |
| *GmWRKY12-qPCR*  *GmWRKY27-qPCR*  *GmWRKY54-qPCR*  *GmNAC11-qPCR*  *GmSIN-qPCR*  *J-qPCR*  *TUB-qPCR*  *EF1β-qPCR*  *GFP-qPCR*  *J-OE* | F: CAATACTGCTGTGGCCGCTA  R: ATCCAGTGTTCGCACCTGTT  F: GTAACAACAGGTTCCAACCGTTCA  R: CTTCTGGTGATTCAGTTTTGGGATT  F: CCCAGTTATGCCTCGCTCAG  R: TGTTGGTGGTGATGGTGCTG  F: CATCATTTAGCTAGCTAGCC  R: TCCGACTTAATCTTTTGATA  F: TTCGCTTCGGGGAATAATCA  R: CTCCATCTTTTGCGGAACTGA  F: CGTGCCTCATAACCGAAGAT  R: CTCGTTACATGACATACTCC  F: TCTTGGACAACGAAGCCATCT  R: GGTGAGGGACGAAATGATCT  F: AAGGACAGAAGACTTGCCACTC  R: GGTGAGGGACGAAATGATCT  F: CCTGAAGTTCATCTGCACCA  R: AAGTCGTGCTGCTTCATGTG  F: CCTAGGATGAAGAGAGGGAAGGATGAT  R: ACGCGTCACTGAGTCATTCTGTTTTCTC |
